# Proliferation and Osteogenic Differentiation of hMSCs on Biomineralized Collagen

**DOI:** 10.3389/fbioe.2020.554565

**Published:** 2020-10-23

**Authors:** Daniel de Melo Pereira, Maria Eischen-Loges, Zeinab Tahmasebi Birgani, Pamela Habibovic

**Affiliations:** Department of Instructive Biomaterials Engineering, MERLN Institute for Technology-Inspired Regenerative Medicine, Maastricht University, Maastricht, Netherlands

**Keywords:** biomineralization, intrafibrillar, osteogenesis, hMSC, bone regeneration, graft substitute

## Abstract

Biomineralized collagen with intrafibrillar calcium phosphate mineral provides an excellent mimic of the composition and structure of the extracellular matrix of bone, from nano- to micro-scale. Scaffolds prepared from this material have the potential to become the next-generation of synthetic bone graft substitutes, as their unique properties make them closer to the native tissue than synthetic alternatives currently available to clinicians. To understand the interaction between biomineralized collagen and cells that are relevant in the context of bone regeneration, we studied the growth and osteogenic differentiation of bone marrow derived human mesenchymal stromal cells (hMSCs) cultured on biomineralized collagen membranes, and compared it to the cell behavior on collagen membranes without mineral. Cells proliferated normally on both biomimetic membranes, and were more triggered to differentiate toward the osteogenic lineage by the biomineralized collagen. This was shown by the elevated mRNA levels of RUNX2, SPP1, ENPP1, and OCN after 3 days of culture, and COL1A1 after 14 days of culture on mineralized collagen. The mRNA levels of the tested markers of osteogenesis were lower on collagen membranes without mineral, with the exception of OCN, which was more highly expressed on collagen than on biomineralized collagen membranes. Expression by hMSCs of OPG, a gene involved in inhibition of osteoclastogenesis, was higher on biomineralized collagen at day 3, while M-CSF, involved in osteoblast-osteoclast communication, was upregulated on both membranes at day 3 and 14 of culture. Alkaline phosphatase activity of hMSCs was high on both biomimetic membranes when compared with cells cultured on tissue culture plastic. Cell-induced mineralization was observed on collagen membranes, while the high mineral content of the biomineralized membranes prohibited a reliable analysis of cell-induced mineralization on these membranes. In conclusion, we have identified that both collagen and biomineralized collagen support proliferation, osteogenic differentiation and mineralization of hMSCs, with biomineralized membranes having a more pronounced positive effect. These findings support the existing evidence that biomineralized collagen is a promising material in the field of bone regeneration.

## Introduction

In the field of bone repair and regeneration, there is a growing need for alternatives to autologous bone transplant as the standard of care. A biomaterial that is able to promote bone growth and regeneration of the injury site, at least as efficiently as autologous bone, is highly desirable. The two main advantages would be increased availability offered by a synthetic biomaterial, and elimination of complications associated with harvesting of bone tissue for transplantation (Calori et al., 2014; Babbi et al., 2016).

A majority of commonly used synthetic bone graft substitutes in the clinic are calcium phosphate (CaP) ceramics, or composites of a CaP ceramic with an organic component, often collagen (Kurien et al., 2013; Tertuliano and Greer, 2016; Baldwin et al., 2019). Such composite biomaterials are similar in composition to the extracellular matrix (ECM) of bone, which consists of about 65 wt% inorganic matrix (hydroxyapatite – HA), 25 wt% organic matrix (mostly collagen type I) and 10% water (Olszta et al., 2007). However, they do not completely replicate the organized hierarchical structure of bone, which, in addition to the composite nature, is known to contribute to the unique mechanical properties of bone (Li and Aparicio, 2013; Reznikov et al., 2018). The structural organization of bone at different length scales comes as a natural source of inspiration for designing materials with potential to become next-generation synthetic bone graft substitutes.

Several attempts have been made to bridge the current gap between synthetic bone graft substitutes and native bone tissue, by replicating the organic-inorganic structure of bone from sub-micrometer to centimeter level (de Melo Pereira and Habibovic, 2018). The replication of the base unit of the bone ECM is of particular interest, as it potentially allows the bottom-up building of larger biomimetic constructs. The building block of bone ECM consists of collagen fibrils with high content (≥65 wt%) of intrafibrillar mineral, specifically nano HA crystals with their c-axis aligned along the collagen fibril length (Olszta et al., 2007). Intrafibrillary mineralized collagen materials can be prepared *in vitro* (Li et al., 2011), via a mineralization method that typically involves one or more charged polymers. These polymers act *in vitro* as analogs of non-collagenous proteins present in the native ECM of bone. They effectively inhibit homogeneous crystallization, i.e., crystallization in solution, forming amorphous CaP-polymer complexes, and promoting mineralization primarily inside the ordered nanostructure of collagen type I fibers (Nudelman et al., 2010, 2012; Habraken et al., 2013).

The potential of biomineralized collagen as the basis for novel bone graft substitutes is evidenced by several studies that investigated this biomaterial in the context of induction of osteogenic differentiation *in vitro* and regeneration of bone defects *in vivo*. A few studies have looked at the extent of osteogenic differentiation of osteoblast-like cell lines (Liu et al., 2014; Wang et al., 2016), mouse (Jiao et al., 2015), or rat MSCs (Wang et al., 2018; Zhang et al., 2018), induced by biomaterials containing biomineralized collagen in their composition. With respect to the application of these materials in the context of bone regeneration, however, more relevant information can be obtained from studies with primary human cells which, though limited in number and using diverse experimental designs and cell types, all have the common denominator of having a scaffold composed of collagen with intrafibrillar mineral and studying the osteogenic differentiation of multipotent cells.

HMSCs were embedded in a collagen gel that was biomineralized with an adapted PILP method, using milk osteopontin as process-directing agent to obtain intrafibrillar as well as extrafibrillar apatite mineral. Gene expression analysis showed that cells within mineralized gels had similar or higher mRNA expression of runt-related transcription factor 2 (RUNX2), osteocalcin (OCN), podoplanin (PDPN) and dentin matrix protein 1 (DMP1), than cells encapsulated in a non-mineralized gel, and moreover that OCN, PDPN, and DMP1 were expressed at levels comparable to or higher than gel-encapsulated cells cultured with osteogenic induction medium. Protein quantification showed that the mineralized collagen gel induced cells to express a higher RANK-L to OPG ratio than either the un-mineralized gel or the osteogenic induction medium, showing the potential for this material to modulate the communication between osteoblast and osteoclast precursors (Thrivikraman et al., 2019).

In a study using periodontal ligament stem cells, increased expression of osteopontin, or secreted phosphoprotein 1 (SPP1), collagen type I (COL1A1), and bone morphogenetic protein 2 (BMP2) was shown after 7 and 14 days of culture on collagen with intrafibrillar mineral versus unmineralized collagen and collagen with extrafibrillar mineral controls. There was also an increased production of mineral nodules (Fu et al., 2016).

Umbilical cord-derived MSCs cultured on scaffolds made of biomineralized collagen showed alkaline phosphatase (ALP) activity similar to cells cultured in the established osteogenic differentiation medium on tissue culture plastic. The scaffolds were implanted in a rabbit femur defect model (Ø8 × 6 mm), showing almost complete healing after 12 weeks (Ye et al., 2016).

A scaffold composed of nano-HA, collagen and poly-L-lactic acid induced osteogenic differentiation of hMSCs, with upregulation of BMP2, COL1A1 and Cathepsin-K (CTSK), shown by microarray analysis (Xu et al., 2016). This biomaterial was also used to repair calcaneal fractures in human patients, where a comparison with autologous bone graft showed no difference in clinical outcome. Seven out of 24 patients had harvest-related complications after 12 months, which is avoidable with the use of synthetic a bone graft substitute (Lian et al., 2013). However, it is not entirely clear whether intrafibrillar mineral was present in this material (Liao et al., 2004). While these few studies show the potential for biomineralized collagen to induce osteogenic differentiation, as well as promising bone defect healing capacity, knowledge about if and how biomineralized collagen triggers osteogenic differentiation is by no means complete. More knowledge of differentiation processes triggered by this biomimetic material and how they relate to osteoinduction *in vivo* is needed to ultimately develop more effective synthetic bone graft substitutes.

In this study, we applied a recognized method for producing biomineralized collagen membranes, and used this material to study the osteogenic differentiation of bone marrow-derived hMSCs. We looked into mRNA expression of early and late markers of osteogenesis and as well as markers involved in osteoblast-osteoclast communication. Furthermore, we characterized the cells regarding their ALP activity and capacity for mineralization.

## Materials and Methods

### Materials

PureCol^®^ collagen type I solution (col-I, 3 mg/mL, 97% bovine dermal type I atelo-collagen) was purchased from Advanced BioMatrix (California, United States, cat# 5005). Poly-L-aspartic acid sodium salt (pAsp, Mw = 27 kDa) was purchased from Alamanda Polymers (Alabama, United States, cat# 000-D200). 1-Ethyl-3-[3-dimethylaminopropyl] carbodiimide hydrochloride (EDC), *N*-hydroxysulfosuccinimide (sulfo-NHS), calcium chloride dihydrate, potassium phosphate dibasic, and all other chemicals were purchased from Sigma-Aldrich (Missouri, United States).

### Preparation of Collagen Membranes

Dense collagen films were prepared in a sterile environment according to the process described by Li et al. (2011), with some modifications. Briefly, the collagen solution, 10 × phosphate buffered saline (PBS) and 0.1 M NaOH were mixed in a volume fraction of, respectively, 0.706, 0.176, and 0.118, yielding a final collagen concentration of 2.1 mg/mL. Gels of 2 mL were formed in up-facing 5 mL syringes with the tip cut off (Sigma-Aldrich, cat# Z248010), at 37°C for 24 h. The syringes containing the gels were inverted on top of nylon meshes of 40 μm pore size (Fischer Scientific, Massachusetts, United States, cat# 11587522) and left at 37°C for 48 h to make the gels loose water under their own weight. After this compression step, syringes were removed, collagen membranes were washed with PBS and cross-linked with 50 mM EDC, 25 mM sulfo-NHS in 50 mM 4-Morpholineethanesulfonic acid (MES) buffer (pH = 7.0), overnight at room temperature. The following day, gels were washed with PBS and incubated with 0.1 M Na_2_HPO_4_ and 2 M NaCl for 2 h, to quench the remaining activated carboxylic acid residues. The membranes were washed three times with PBS for 2 h. Membranes were then mineralized or kept in PBS at 4°C for maximum 1 week.

### Mineralization of Collagen Membranes

Mineralization solution was prepared according to the polymer-induced liquid precursor (PILP) method (Li et al., 2011). Briefly, stock solutions of calcium (9 mM CaCl_2_) and phosphate (4.2 mM K_2_HPO_4_) were prepared in a buffer with 50 mM TRIS base, 150 mM NaCl, with a pH of 7.8 at room temperature. Prior to incubation, pAsp was added to the calcium solution, mixed and let rest for 5 min, followed by addition of the same volume of phosphate precursor solution. The final concentration of pAsp was 100 μg/mL. After mixing the two precursor solutions, the col-I membranes were added (40 mL of solution was used per membrane) and incubated in a water bath at 37°C for 7 days. Before cell culture, membranes were punched with a 10 mm metal puncher for culture in 48-well plates. Collagen or biomineralized collagen membranes were washed with PBS and incubated in cell culture medium for 3–4 h, prior to cell culture. All steps were performed in a sterile environment.

### Cell Culture

Human mesenchymal stromal cells were isolated from bone marrow aspirates (Booth et al., 2007; Fernandes et al., 2010) obtained from one donor, who has given written informed consent. After isolation, the cells were seeded at a density of 1,500 cells/cm^2^ in tissue culture T-flasks, and expanded in growth medium (GM), consisting of α-MEM without nucleotides and with Glutamax^TM^ (Thermo Fisher Scientific, cat# 32561), supplemented with 10 v/v% fetal bovine serum (Sigma-Aldrich, cat# F7524, batch# BCBT6987), and 20 mM ascorbic acid (Sigma-Aldrich, cat# A8960). Osteogenic differentiation medium (OM) was prepared by supplementing GM with 100 nM dexamethasone (Sigma-Aldrich, cat# D8893). Mineralization medium (MM) was prepared by supplementing OM with 10 mM β-glycerophosphate (Sigma-Aldrich cat# 50020). Cells were cultured in standard conditions of 37°C in a humidified atmosphere with 5% CO_2_, and medium was replaced every 2 or 3 days. Upon reaching 70–80% confluence, the cell layer was washed with warm PBS and detached using a 0.05% trypsin-EDTA solution (Thermo Fisher Scientific, cat# 253000) until detachment was visible under a light microscope, up to a maximum of 5 min. Trypsin was neutralized with addition of GM, the cell suspension was centrifuged at 300 rcf for 5 min and re-suspended in GM. Cells were counted with a haemocytometer and dilution of the cell suspension was made according to the seeding densities required.

For quantification of DNA and ALP activity, which were performed on the same set of samples, cells were seeded at a density of 10.000 cell/cm^2^ in 48-well plates on collagen or biomineralized collagen, with tissue culture plastic (TCP) as a control. Cells on TCP were cultured in GM or OM, while on both types of membranes, the culture was performed in GM only. There were six replicates per condition. At each time point (3, 7, 14, and 21 days), medium was aspirated and samples washed with warm PBS. All PBS was removed and the plate was kept at −80°C until further analysis.

The same seeding and sample handling was performed for the quantitative real-time polymerase chain reaction (qRT-PCR) experiment, at 3, 7, and 14 days, also with 6 replicates per condition.

### DNA Quantification

Total content of nucleic acid, i.e., cell DNA, per sample was quantified with the Cyquant^TM^ kit (Thermo Fisher Scientific, cat# C7026) following the manufacturer’s instructions. Briefly, frozen plates were thawed and collagen and mineralized collagen membranes were transferred to new plates. Lysis buffer from the kit was added to each well (300 μL) and two more freeze-thaw cycles were done. Subsequently, samples were placed on ice in a ultrasonic water bath for 30 min. Cell lysates were collected and transferred to micro centrifuge tubes, spun down for 10 s and 100 μL of the supernatant transferred to clear bottom black 96-well plates, with technical duplicates. The dye-containing buffer was added (2× concentrated, 100 μL) and plates were incubated at room temperature for 5 min in the dark. Fluorescence measurements were made with a CLARIOstar Plus microplate reader (BGM Labtech, Germany) with excitation and emission wavelength of 485 ± 10 and 530 ± 10 nm, respectively. A standard curve of known cell numbers vs. fluorescence intensity was prepared from the same cell suspension used in the experiment, i.e., 11, 22, 33, 44, and 110k cells in 48-well plate, frozen after 6 h in culture. Total cell number per condition was calculated by subtracting the blank fluorescence reading, i.e., dye-containing lysis buffer, from each measurement, and converting fluorescence to cell number with the standard curve. The results are presented as mean and standard deviation of the biological replicates.

### Quantitative Real-Time Polymerase Chain Reaction (qRT-PCR)

Samples were collected in TRIzol Reagent (Thermo Fisher Scientific). Extraction of RNA was performed using the phenol-chloroform method and purification was done using the RNeasy Minikit (QUIAGEN, Germany), in accordance with the manufacturer’s recommendations. The extracted RNA of biological replicates were pooled in groups of 2, in order to increase the RNA quantity in each sample, resulting in 3 replicates per condition. RNA purity and quantity were determined using a BioDrop μLITE instrument (BioDrop, United Kingdom). For each sample, 250 ng of RNA were reverse transcribed into cDNA using iScript cDNA Synthesis Kit (Bio-Rad, CA, United States) following manufacturer’s instructions. Amplification of cDNA (20 ng) by qRT-PCR was performed on a CFX96 Real-Time PCR Detection System (Bio-Rad) using the iQ SYBR Green Supermix for qPCR (Bio-Rad). Transcription levels of osteogenic biomarkers including runt-related transcription factor 2 (RUNX2), SPP1, ALP, COL1A1, osteocalcin (OCN), Ectonucleotide Pyrophosphatase/Phosphodiesterase 1 (ENPP1) as well as osteoclast modulatory biomarkers including receptor activator of nuclear factor kappa-B ligand (RANKL), osteoprotegerin (OPG) and macrophage colony-stimulating factor (MCSF), were determined. Primer sequences for each marker can be found in Table 1. Fold expression values were determined using ΔΔ*C*t method after normalizing each target gene with respect to the housekeeping gene (GAPDH) and to the expression of the target gene in hMSCs at day 3 on culture plates in GM.

**TABLE 1:** Primer sequences for qRT-PCT.

**Gene**	**Forward (5′–3′)**	**Reverse (5′–3′)**
RUNX2	CCGCCTCAGTGATTTAGGGC	GGGTCTGTAATCTGACTCTGTCC
SPP1	GGTGATGTCCTCGTCTGTA	CCAAGTAAGTCCAACGAAAG
ALP	ACAAGCACTCCCACTTCATC	TTCAGCTCGTACTGCATGTC
COL1A1	GAGGGCCAAGACGAAGACATC	CAGATCACGTCATCGCACAAC
OCN	TGAGAGCCCTCACACTCCTC	CGCCTGGGTCTCTTCACTAC
ENPP1	CAAAGGTCGCTGTTTCGAGAG	TGCACGTCTCCTGGTAATCTAAA
RANKL	CAACATATCGTTGGATCACAGCA	GACAGACTCACTTTATGGGAACC
OPG	CACAAATTGCAGTGTCTTTGGTC	TCTGCGTTTACTTTGGTGCCA
MCSF	AGACCTCGTGCCAAATTACATT	AGGTGTCTCATAGAAAGTTCGGA

### Quantification of ALP Activity

Alkaline phosphatase activity was quantified with the CDPStar^®^ reagent (Sigma-Aldrich, cat# GERPN3682). 10 μl of cell lysate supernatant (as described for quantification of total cell number) were transferred, with technical duplicates, to a 96-well plate. 40 μL of CDPStar reagent was added to each well, followed by 30 min incubation at room temperature in dark. Luminescence intensity was read with a CLARIOstar Plus microplate reader. The results were normalized to the cell number, and are presented as mean and standard deviation of the biological replicates.

### Scanning Electron Microscopy (SEM) and Energy-Dispersive X-Ray Spectroscopy (EDS)

The morphology and chemical composition of the membranes were characterized using an SEM coupled with an EDS detector. Collagen and biomineralized collagen membranes were dehydrated using a sequence of, first, mixtures of PBS and ethanol (70:30, 60:40 and 50:50) for 15 min each; and second, mixtures of distilled water and ethanol (40:60, 30:70, 20:80, 10:90, and 100% ethanol) for 15 min each. This was followed with a 30 min incubation in a 50:50 mixture of ethanol and hexamethyldisiloxane (HMDS), followed by 30 min of incubation in HMDS. The last step consisted of removing the HMDS and leaving the membranes to dry overnight. Dried membranes were glued to aluminum stubs with carbon tape and silver paint. Samples were sputter coated with a 2 nm iridium layer for increased conductivity using a Q150TES sputter coater (Quorum, United Kingdom).

For observation of cell-induced mineralization, membranes with cells cultured on them were washed with warm PBS following cell culture medium aspiration, and fixed with warm 4% formaldehyde in PBS, at room temperature for 15 min. After washing with PBS, the samples were dehydrated and prepared for SEM as described above.

Samples were imaged with a TENEO electron microscope (FEI, OR, United States) operating in Optiplan mode at 2–5 kV and 2–10 mm working distance, using the T1 in-column, ETD or back-scatter detectors. For EDS analysis, samples were imaged with a VERSA electron microscope (FEI) equipped with an EDS detector (EDAX, NJ, United States), operating at 10 kV and 10 mm working distance.

### Statistical Analysis

Statistical testing of mean differences was performed in GraphPad Prism (version 8.3) using two-way analysis of variance (ANOVA) of independent samples, looking at differences between conditions within each timepoint. A Bonferroni *post hoc* test was used to correct for multiple comparisons (one family for all comparisons). Mean differences were considered statistically significant for *p*-value < 0.05. Data in all figures is presented as mean and standard deviation, unless otherwise specified, and significance is denoted as (*) *p* < 0.05, (**) *p* < 0.01, (***) *p* < 0.001, (****) *p* < 0.0001.

## Results

### Characterization of Collagen and Biomineralized Collagen Membranes

**FIGURE 1:**
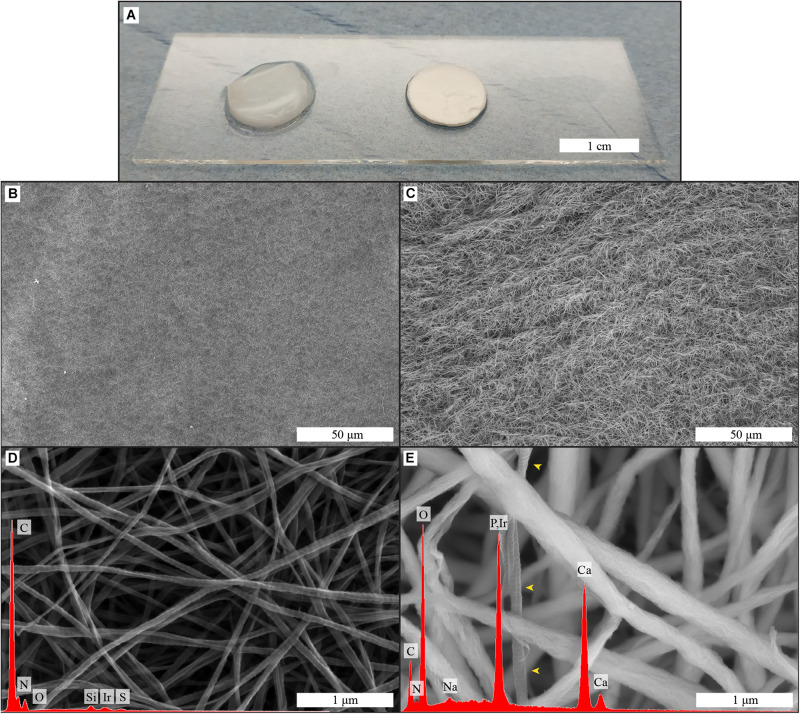
Macroscopic image **(A)** of collagen and biomineralized collagen as used for cell culture experiments; surface structure and elemental composition of collagen **(B,D)** and biomineralized collagen **(C,E)** membranes, obtained by SEM-EDS. Low magnifications images of the surface of collagen **(B)** and biomineralized collagen **(C)**. High magnification image of collagen membrane with corresponding EDS spectrum **(D)**, collagen banding is visible and there is no calcium or phosphorus signal. High magnification image of biomineralized collagen and corresponding EDS spectrum **(E)**, where thicker fibers are visible, and no banding is discernible, expect in one fiber that is not mineralized (yellow arrowheads). Both types of membrane show fibrillar structure.

The morphology and elemental composition of both biomimetic membranes was analyzed by SEM-EDS, and the results are displayed in Figure 1. The membranes as used for cell culture are shown in Figure 1A.

The collagen membranes were compact films consisting of randomly oriented collagen fibers, with even surface morphology exemplified by the images in Figures 1B,C. Fibers of the collagen matrix had an average diameter of 60 ± 13 nm and displayed a banding pattern along their length (Figure 1D). The fibrous structure remained after the mineralization process, although the fibers were wider, 201 ± 56 nm (Figure 1E), and the banding pattern was no longer visible. Elemental analysis of both membranes showed that calcium and phosphorus was only present in biomineralized collagen membranes (Figures 1D,E).

### Effect of Collagen and Biomineralized Collagen on Proliferation of hMSCs

**FIGURE 2:**
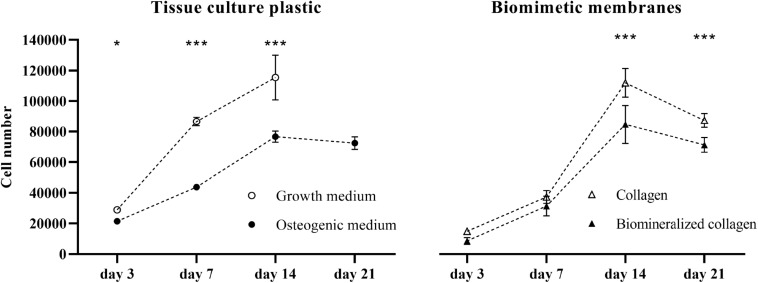
Proliferation of hMSCs on tissue culture plastic in GM and OM as well as on the biomimetic collagen and biomineralized collagen membranes in GM. Cell proliferation is expressed as cell number at 3, 7, 14, and 21 days of culture. The data point for 21 days in the GM condition is missing, due to cells becoming over-confluent and eventually detaching from the tissue culture plate. Data are represented as mean ± SD (*n* = 6). Data is presented as mean and standard deviation and significance is denoted as *p < 0.05, ***p < 0.001.

To explore the effect of collagen and mineralized collagen on the proliferation of hMSCs, using TCP as a control, DNA content of the cells on membranes was quantified and converted to cell number (Figure 2). Cells cultured on TCP in GM exhibited a steady increase in number until day 14. Beyond this time point, the cells were detached from the TCP due to overgrowth of the cell monolayer in the culture well, and hence, no measurement could be done for GM. hMSCs cultured in OM were proliferating at a lower rate than in GM at days 7 and 14, and no further growth in OM was observed between day 14 and 21. Cells cultured on collagen membranes showed a lower proliferation than on TCP in GM at days 3 and 7. A boost in proliferation was observed between day 7 and 14 on collagen membranes where cells reached the same cell number as on TCP in GM. No further increase in cell number was observed on collagen membranes at day 21. hMSCs cultured on mineralized collagen had a lower cell number than cells on TCP in GM at days 3, 7, and 14. No further growth was observed at day 21. On both collagen and mineralized collagen, cells followed a proliferation pattern similar to that on TCP in OM until day 7 of culture. At days 14 and 21, proliferation was increased on collagen membranes whereas cells on mineralized collagen maintained a proliferation similar to the cells cultured on TCP in OM. Taken together, both collagen and mineralized collagen supported proliferation of hMSCs, with, at the later time points of 14 and 21 days, a higher cell number on collagen than on mineralized collagen.

### Osteogenic Gene Expression of hMSCs on Biomimetic Membranes

**FIGURE 3:**
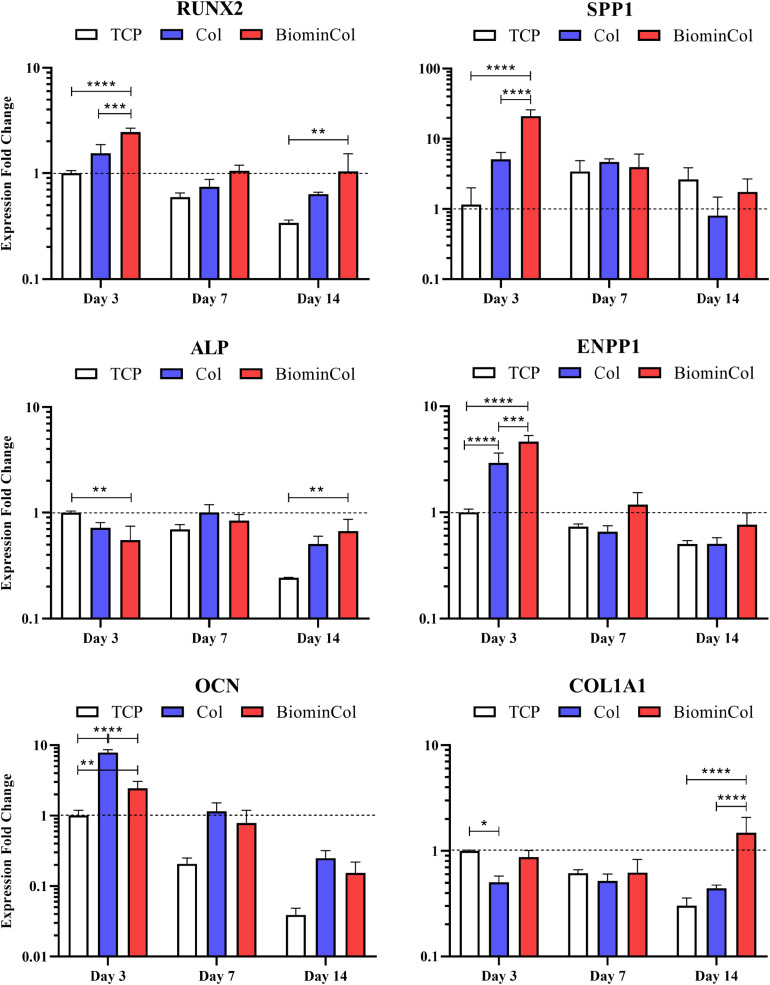
Gene expression profiles for RUNX2, SPP1, ALP, ENPP1, OCN, and COL1A1 of hMSCs cultured on tissue culture plastic (TCP) control, collagen (Col), and biomineralized collagen (BiominCol) for 3, 7, and 14 days. Data are represented as mean ± SD (*n* = 3). Data is presented as mean and standard deviation and significance is denoted as *p < 0.05, **p < 0.01, ***p < 0.001, ****p < 0.0001.

Osteogenic differentiation of hMSCs was analyzed by quantifying the expression of osteogenic biomarkers at the mRNA level via qRT-qPCR (Figure 3). The selected markers were RUNX2, a transcription factor associated with activation of osteogenic genes (Ducy et al., 1997) and SPP1, a non-collagenous protein with roles in cell attachment and matrix mineralization (Komori, 2006). In addition, markers for two matrix proteins were assessed, COL1A1, the main constituent of the organic part of the matrix (Quarles et al., 1992) and OCN, which is involved in matrix mineralization by binding hydroxyapatite (Tsao et al., 2017), as well as two other markers involved in the regulation of inorganic phosphate, ALP and ENPP1 (Hessle et al., 2002; Johnson et al., 2003).

RUNX2, an early transcription factor (Ducy et al., 1997), was significantly upregulated at day 3 on collagen (1.6×) as well as on mineralized collagen (2.5×), compared with TCP. After 7 days of culture, a similar trend was observed, where a higher expression on both materials was detected as compared to the control, i.e., TCP, with the highest expression on biomineralized collagen. At day 14, when cells on both types of biomimetic membrane showed higher expression levels than the control, i.e., cells cultured on TCP, RUNX-2 expression on biomineralized collagen was significantly upregulated by 3.1×. When comparing collagen and mineralized collagen, a trend of a higher RUNX2 expression on mineralized collagen was observed at each time point.

A significant upregulation of SPP1 on biomimetic membranes as compared to TCP was detected at day 3, with a 4× increase on collagen and the highest increase of 18× on mineralized collagen, following a behavior similar to RUNX2. After 7 days, no differences in expression of SPP1 were observed between conditions and, after 14 days, a slight decrease in expression relative to the control was observed on collagen, without statistical significance, whereas cells on mineralized collagen showed similar expression levels as the control. A decrease in expression over time was observed on mineralized collagen, while expression for TCP slightly increased from days 3 to 7 and were maintained at day 14. The expression overtime on collagen was unchanged at 3 and 7 days and showed a slight decrease at 14 days.

Expression of ALP, which plays a role in matrix mineralization (Hessle et al., 2002; Johnson et al., 2003), was slightly downregulated early, at day 3, on collagen relative to the control, as well as on mineralized collagen with a 2× decrease. Comparable expression levels between all conditions were observed at day 7. After 14 days, the expression on collagen and mineralized collagen was increased as compared to TCP, with a 2.7× statistically significant increase on mineralized collagen. ALP expression was downregulated in the TCP control overtime but collagen and mineralized collagen maintained the same level overtime.

Expression of ENPP1, an enzyme that regulates bone mineralization (Hessle et al., 2002; Johnson et al., 2003), was evaluated. Both ENPP1 and ALP are involved in maintaining bone mineralization in equilibrium. Bone mineralization relies on the availability of inorganic phosphate (Pi), which together with calcium crystallizes to form HA. ALP hydrolyses inorganic pyrophosphate (PPi) to generate Pi, which promotes mineralization, and ENPP1 generates PPi, which antagonizes mineralization. A statistically significant upregulation of ENPP1 was observed on both collagen (3×) and mineralized collagen (4.6×) relative to the control, at day 3. A significant increase of 1.6× was observed on mineralized collagen as compared to collagen. A pronounced decrease was observed at day 7 on collagen and mineralized collagen, as well as a smaller decrease on TCP. This trend was maintained after 14 days with a slight increase of expression on biomineralized collagen, without statistical significance.

Osteocalcin expression was significantly upregulated on collagen, being 3× higher than on mineralized collagen and 8× higher as compared to TCP. The expression on mineralized was also significantly increased (2.4×) as compared to the control. This trend of highest expression on collagen, followed by mineralized collagen and the lowest being on TCP, was maintained at days 7 and 14, although the difference of expression between collagen and mineralized was not as pronounced as at the earliest time point. A decrease of OCN expression was observed over time for all substrates.

COL1A1, the main constituent of the organic matrix and therefore an indicator of ECM deposition (Quarles et al., 1992), was significantly downregulated on collagen at day 3, by half relative to the control, expression on mineralized collagen was similar as on TCP. No differences between conditions was observed at day 7. At day 14 on mineralized collagen, COL1A1 expression increased significantly by 5× relative to TCP and by 3.4× relative to collagen. A decreasing trend over time was observed on TCP.

### Expression of Osteoclast-Related Genes by hMSCs on Biomimetic Membranes

**FIGURE 4:**
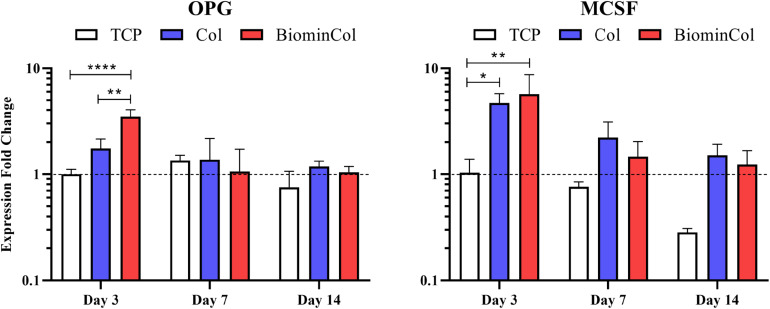
Gene expression profiles for OPG and MCSF of hMSCs cultured on tissue culture plastic (TCP) control, collagen (Col), and biomineralized collagen (BiominCol) for 3, 7, and 14 days. Data are represented as mean ± SD (*n* = 3). Data is presented as mean and standard deviation and significance is denoted as *p < 0.05, **p < 0.01, ****p < 0.0001.

Potential modulation of osteoclast behavior by hMSCs cultured on collagen or biomineralized collagen was analyzed by assessing the expression of factors involved in osteoclastogenesis, including OPG, MCSF and RANKL (Lees and Heersche, 1999; Lee et al., 2010) at mRNA level (Figure 4). RANKL expression was below the detection limit of the qRT-PCR, indicating a very low expression level in all samples (Supplementary Figure 1). OPG expression in hMSCs on TCP was constant over time. An early upregulation at day 3 was observed on collagen, which was not statistically significant. An increase was also observed on mineralized collagen with a 3.5× increase relative to TCP and 2× increase relative to collagen, which were both statistically significant. At days 7 and 14, expression levels on collagen and mineralized collagen decreased to levels similar to those on TCP. MCSF expression at day 3 was significantly increased 4.5× on collagen and 5.5× on mineralized collagen relative to TCP. This trend, of a higher expression on both biomimetic membranes, was maintained at days 7 and 14. Overall, however, MCSF expression decreased over time, from day 3 until day 14, on all substrates.

### ALP Activity

**FIGURE 5:**
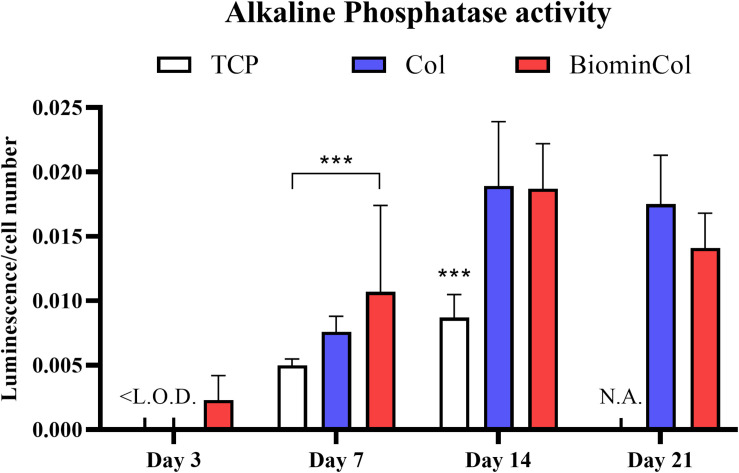
Alkaline phosphatase (ALP) activity of hMSC, normalized to cell number. Cells were cultured on tissue culture plastic (TCP) control, collagen (Col), and biomineralized collagen (BiominCol) for 3, 7, 14, and 21 days. Data are represented as mean ± SD (*n* = 6). Data under the limit of detection are denominated as <L.O.D (growth medium and collagen at day 3), and missing data is labeled as N.A. (TCP control at day 21). Data is presented as mean and standard deviation and significance is denoted as ***p < 0.001.

In addition to assessing the mRNA expression levels of ALP, its activity was also analyzed as biochemical marker for osteoblast-like function in hMSCs undergoing osteogenic differentiation on different materials (Figure 5). ALP activity of hMSCs after 3 days was below the limit of detection for all conditions except the biomineralized collagen. On day 7, ALP activity was around two times higher on the biomineralized collagen than on the TCP control. Cells on mineralized collagen also showed a higher ALP activity than on collagen, although this difference was not statistically significant. There was a general increase from 7 to 14 days, and hMSCs cultured on both biomimetic membranes showed approximately twofold higher ALP activity than those cultured on TCP, while no significant effect of collagen mineralization was observed. Finally, on day 21, no measurement could be done on the TCP control, as cells grew over-confluent and samples had to be discarded. Collagen and biomineralized collagen showed comparable ALP activity on day 21, with a slight decrease compared to day 14.

### Cell-Induced Mineralization

**FIGURE 6:**
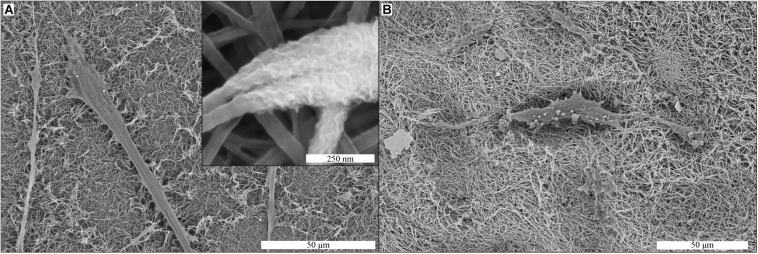
Cell-induced mineralization on collagen **(A)** and biomineralized collagen **(B)** membranes, shown by SEM imaging. Cells were cultured on membranes for 21 days in MM. Mineral deposits are visible in the collagen membrane (inset A). These deposits were not observed on biomineralized collagen **(B)**.

Production of mineralized ECM by the hMSCs was evaluated by SEM and EDS analyses of membranes after 21 days of cell culture in MM. Mineral deposits produced by hMSCs were observed on collagen membranes (Figure 6A). The mineral deposits were found as a layer surrounding the collagen fibers of the membrane (inset Figure 6A), in close proximity to the cells on the membrane. These deposits were also found in the areas of the collagen membrane where no cells were observed. On mineralized collagen membranes, the only possible indication of cell-induced mineralization were spherical structures with the diameter of a few micrometers, which were observed in close association with cell filopodia (Figure 6B).

**FIGURE 7:**
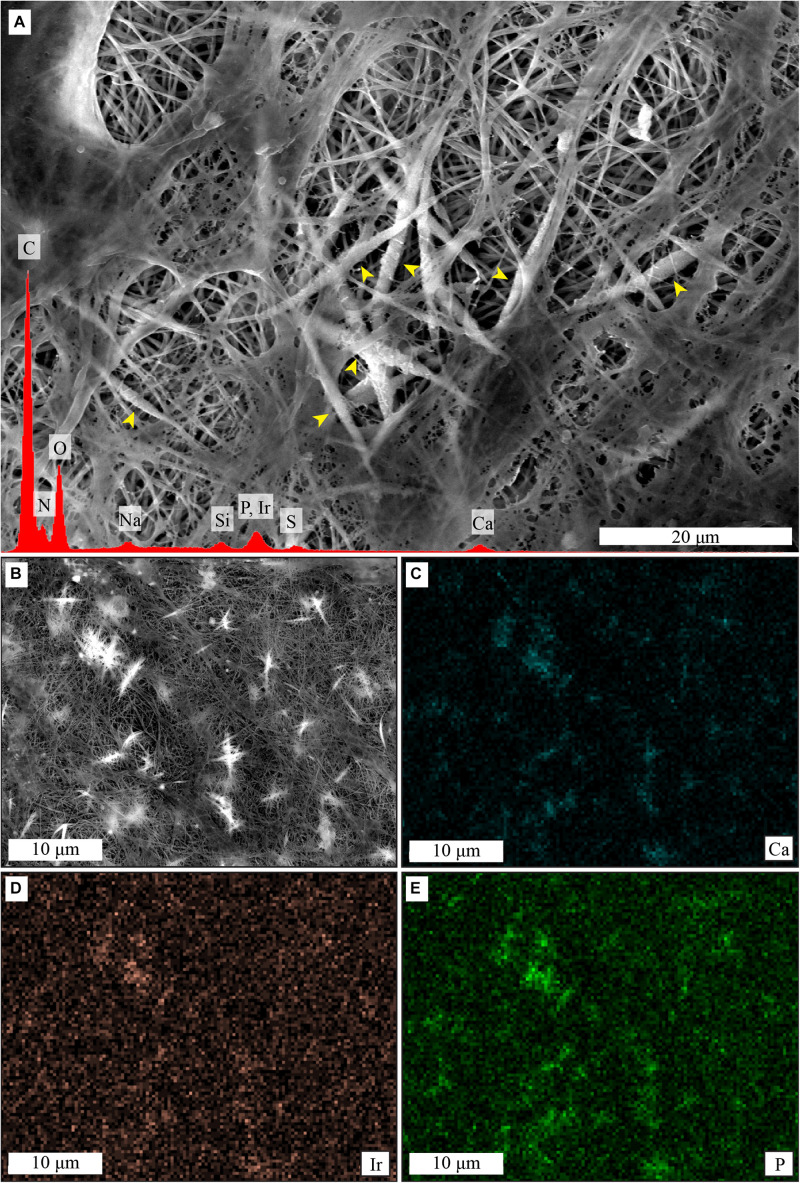
Cell-induced mineralization on collagen membranes, shown by SEM imaging and EDS analysis. Mineral deposits are visible as thicker and whiter fibers in the center of the image **(A)**, yellow arrowheads. EDS spectra of **(A)**, in red, shows presence of calcium and phosphorus. ESD mapping **(B–E)** shows that signal from calcium **(C)** and phosphorus **(E)** comes predominantly from the thicker and whiter mineral deposits that surround the collagen fibers, as seen in the previous figures. In contrast, the signal from iridium **(D)** is more homogeneous.

Elemental analysis by EDS showed presence of calcium and phosphorus on the collagen membranes after cell culture in MM (Figure 7A), and elemental mapping showed that the signal was originating from the mineral deposits (Figures 7B–E). No mineral deposits were observed by SEM and no calcium or phosphorus presence was detected by EDS when hMSCs were cultured on collagen membranes in GM for 21 days (Supplementary Figure 2). Similarly, no evidence for mineral deposits was found when collagen membranes were incubated in MM for 14 days in the absence of cells (Supplementary Figure 3).

## Discussion

The intrinsic self-healing capacity of bone falls short in repairing critical-sized bone defects. To avoid drawbacks commonly associated with using bone grafts, such as limited availability as well as donor site morbidity (Calori et al., 2014; Babbi et al., 2016), synthetic bone graft substitutes are in demand as replacement for autologous bone (Kurien et al., 2013; Baldwin et al., 2019). Intrafibrillary biomineralized collagen is a promising biomaterial for bone regeneration applications, as it mimics not only the organic-inorganic composition, but also the structure of the ECM of bone tissue at the sub-micron scale. In this study, we aimed to complement the existing knowledge on the relevance of biomineralized collagen in bone regeneration, by evaluating the behavior of clinically-relevant hMSCs on intrafibrillarly mineralized collagen membranes in comparison with collagen membranes without mineral. The differentiation of undifferentiated stem cells into osteogenic progenitors, i.e., osteoinduction, is a key process in regeneration of bone defects (Bohner and Miron, 2019) and gaining further insight into this process is a valuable resource for designing novel and more functional bone graft substitutes. There are only a few *in vitro* studies exploring the effects of biomineralized collagen on osteogenic differentiation of human stem cells (Fu et al., 2016; Xu et al., 2016; Ye et al., 2016; Thrivikraman et al., 2019). The results with hMSCs described here contribute to the growing evidence that this biomaterial is a promising candidate for developing synthetic bone graft substitutes.

Biomineralized collagen membranes, consisting of collagen type I containing intrafibrillar CaP mineral, were prepared following the established PILP route (Li et al., 2011). Dense type I collagen membranes had a fibrillar structure with typical D-banding characteristic of native collagen, a requirement for intrafibrillar mineralization to occur (Olszta et al., 2007). Upon mineralization of the membranes, the fibrillar structure of the membrane was preserved. Typical crystal deposition from saturated Ca^2+^ and PO_4_^3–^ solutions (Costa et al., 2012) was not observed on the surface of the material, while the elemental analysis showed that CaP was present in abundance. These observations indicate that CaP was present in the mineralized collagen membrane, but not on the surface of the fibers, and therefore must reside within the collagen fibers. Further evidence of this is the observed increase in fiber diameter after the biomineralization process, being roughly three times the size of the collagen membrane fibers. We therefore conclude that homogenous crystallization, i.e., crystallization in solution, did not occur, and mineral was instead deposited within the collagen fibrils, which is expected when a polyanion such as pAsp is added to the mineralization process, and is in accordance with previous studies in which PILP was used for collagen biomineralization (Jee et al., 2010; Thula et al., 2011).

Mineralized collagen membranes were used to study proliferation and differentiation of hMSCs at the mRNA level, using collagen membranes without mineral as a control. Moreover, ALP activity and mineralization of the cells were analyzed.

Both biomimetic membranes with and without mineral supported the growth of hMSCs over a period of 21 days, with mineralized collagen showing a somewhat lower proliferation rate, in particular at the later time points of 14 and 21 days. Previous studies have also shown that collagen matrices with and without CaP mineral support cell proliferation. For example, in a study with MC3T3-E1 osteoblasts, a more pronounced proliferation on collagen without than on collagen containing mineral was observed, however, this analysis was only performed over a period of 7 days (Wang et al., 2016). Collagen with and without HA supported proliferation of rat MSCs over a period of 14 days without significant differences between the materials (Sun et al., 2018), whereas periodontal ligament stem cells were present in higher numbers on intrafibrillarly mineralized collagen than on the collagen control at 3, but not at 7 days of culture (Fu et al., 2016).

Regarding osteogenic differentiation, in general, hMSCs showed an upregulation of osteogenic differentiation markers when cultured on mineralized collagen as compared to collagen without mineral.

RUNX2, an essential transcription factor required for determination of the osteoblastic lineage in hMSC at an early stage (Ducy et al., 1997) as well as SPP1, another early marker of osteogenesis, that has been shown to be expressed in immature osteoblasts (Komori, 2006), were enhanced at an early time point on biomineralized collagen membranes. This effect on RUNX2 was maintained, however, at lower mRNA levels and decreased for SPP1 over time. We observed a similar trend in time on collagen, however, the expression of RUNX2 was always lower than on biomineralized collagen indicating a lower degree of osteogenic differentiation. An early upregulation of RUNX2, followed by a slow decrease, is a pattern found over the course of osteogenic differentiation of hMSCs *in vitro* (Shekaran et al., 2015). RUNX2 is regulated by several upstream pathways, which are involved in osteogenic differentiation, including BMP and WNT signaling pathways (Rutkovskiy et al., 2016). Its upregulation indicates an activation of these pathways and, hence, material-induced osteogenic differentiation. This also explains the upregulation of SPP1, which is a direct target of RUNX2 signaling (Ducy et al., 1997). SPP1 is an important factor of matrix mineralization, which also plays a role in many other functions such as cell survival, migration, regulation of inflammation and angiogenesis (Giachelli and Steitz, 2000).

ALP and ENPP1 are known to positively and negatively regulate matrix mineralization in native bone (Hessle et al., 2002; Johnson et al., 2003), respectively. The mRNA level of ALP was lower, while the ENPP1 mRNA was higher on biomineralized collagen compared to the control at day 3. The opposite trend in ALP levels overtime was observed, with cells cultured on TCP showing a significant decrease, while biomineralized collagen maintained the level comparable to that at day 3. A substantial decrease of overall ENPP1 expression was observed at later time points, plausibly indicating the onset of matrix mineralization. ALP and ENPP1 expression of cells cultured on the collagen membranes and biomineralized collagen were similar, with ENPP1 expression being slightly lower in the absence of mineral. ENPP1 expression has previously been shown to be upregulated on CaP ceramics with intrinsic osteoinductive potential (Othman et al., 2019). To our knowledge, this is, however, the first time that ENPP1 expression has been investigated on collagen and biomineralized collagen.

OCN, the most abundant non-collagenous protein in bone (Tsao et al., 2017), was surprisingly expressed at an early stage with an upregulation on biomineralized collagen compared to the control. This trend was maintained over time and a similar effect on OCN expression was observed on collagen membranes without mineral. OCN has been shown to play an important role in osteoblast maturation, inter alia, promoting matrix mineralization by binding to HA (Hauschka and Wians, 1989). A study by Tsao et al. (2017) showed that low OCN levels potentially lead to a delayed matrix mineralization. An early expression of OCN as observed here may indicate that biomimetic collagen membranes are able to accelerate osteogenic differentiation, however, further research is needed to provide conclusive evidence for this.

COL1A1, the main component of the organic matrix, was higher at day 14 on biomineralized collagen relative to TCP (3.4×) and collagen (4.9×), suggesting matrix deposition by cells of the osteoblastic lineage (Quarles et al., 1992). In contrast, collagen membranes without mineral were unable to stimulate COL1A1 expression, which was downregulated early on and kept at baseline until later time points. This effect of pure collagen scaffolds has previously been observed in different cell types (Fu et al., 2016; Sun et al., 2018).

In a study by Sun et al. (2018), osteogenic differentiation of rat MSCs on collagen versus a collagen/HA composite, without intrafibrillar mineral, was tested, showing the results consistent with those presented here. Cells cultured on collagen/HA scaffolds showed, overall, the highest osteogenic differentiation capacity, supported by a rapid activation of RUNX-2 and the high mRNA levels of ALP and especially COL1A1. The authors attributed these effects to the differences in microstructure and stiffness of the materials (Sun et al., 2018). An upregulation of RUNX2 was also observed on CaP ceramic controls without collagen and may therefore also be influenced by the presence of CaP and/or free calcium and inorganic phosphate ions.

mRNA expression of ALP in hMSCs cultured on collagen and mineralized collagen has been sparsely investigated. However, a study investigating osteogenic differentiation of hMSCs on nanoparticulate (mineralized) collagen glycosaminoglycan scaffolds showed a low ALP expression at day 3 and a slight increase at day 7 (Ren et al., 2016). Both scaffolds with and without mineral also showed an upregulation in matrix markers, such as OCN and COL1A1, as compared to the beginning of culture (day 0), which is in accordance with our results. In a study by Fu et al. (2016), osteogenic differentiation of human periodontal ligament stem cells was evaluated on collagen, intrafibrillarly mineralized collagen and extrafibrillarly mineralized collagen. Cells cultured on intrafibrillarly mineralized collagen showed an increased expression of osteogenic genes, such as SPP1, COL1A1, and BMP-2 as compared to collagen at days 7 and 14.

In the context of bone regeneration, not only the osteoblast function, as the bone-forming unit, but also the osteoclast function, the bone-resorbing unit, is of importance. In native bone, these two processes are in balance, maintaining a constant, homeostatically-controlled amount of bone ECM (Rodan, 1998). Therefore, in this study, we took first steps in analyzing how biomimetic membranes may influence osteoblast-osteoclast communication by assessing the mRNA levels of the biomarkers involved in this cellular crosstalk in hMSCs.

The RANKL/OPG ratio has an important role in osteoclast regulation. RANKL activates osteoclasts by binding to its membrane-bound receptor RANK, and OPG has an inhibitory effect by binding RANKL to prevent osteoclast activation (Lee et al., 2010). As reviewed by Kapasa et al. (2017), previous research indicated that changes in RANKL/OPG balance might reduce implant complications, which are caused by an increased osteoclastogenesis accompanied by chronic inflammation in which significantly elevated levels of RANKL are frequently observed. This results in poor osseointegration of the implant, prosthetic loosening and non-union of the defect. Here, low mRNA levels of RANKL in hMSCs were detected overtime. Osteoblasts can exhibit a pro-osteoclastic phenotype, expressing RANKL. This was previously observed in an immature subpopulation of osteoblasts and simulated *in vitro* by the stimulation with osteotropic factors vitamin D3, dexamethasone or their combination (Atkins et al., 2003). Here, however, low mRNA levels of RANKL were detected overtime. Ren et al. (2019) combined OPG adenoviral expression in hMSCs with nanoparticulate mineralized collagen glycosaminoglycan scaffolds or non-mineralized scaffolds and observed a decreased RANKL/OPG ratio. RANKL expression was detected not only in OPG transfected hMSCs but also in non-transfected hMSCs on mineralized and non-mineralized material. The major difference in Ren et al. (2019)’ culture protocol compared to ours was the culture of hMSCs in osteogenic medium containing dexamethasone. We speculate that the lack of dexamethasone in our cultures, chosen to isolate the effect on the biomaterials from other stimulators of osteogenic differentiation, may be the reason for the low RANKL expression observed.

OPG mRNA expression, on the other hand, was upregulated in hMSCs cultured on biomineralized collagen indicating the potential of this biomaterials to modulate the osteoblast-osteoclast crosstalk.

Jiao et al. observed that mouse MSCs cultured on biphasic silica/apatite intrafibrillarly mineralized collagen scaffolds expressed higher mRNA levels of OPG (fivefold increase), and lower mRNA levels of RANKL relative to collagen scaffolds without mineral (Jiao et al., 2015). OPG expression on intrafibrillarly calcified collagen scaffolds without silica was only slightly increased and no difference in RANKL expression relative to collagen without mineral was observed. This indicated an inhibitory effect on RANKL-mediated osteoclastogenesis induced by biphasic silica/apatite intrafibrillarly mineralized collagen. This hypothesis was confirmed in a follow up experiment in which osteoclasts were exposed to conditioned medium from hMSCs cultured on these scaffolds, finally resulting in decreasing numbers of functional osteoclasts, i.e., tartrate-resistant acid phosphatase (TRAP)-positive cells.

In our study, we additionally observed an upregulation of MCSF mRNA expression, which has been suggested to activate osteoclastic bone resorption (Lees and Heersche, 1999), in hMSCs cultured on both collagen and biomineralized collagen membranes, with the effect remaining elevated compared to control, yet overall, decreasing overtime.

Taken together, our data suggests that collagen and biomineralized collagen can potentially modulate osteoclastogenesis and the osteoclast-osteoblast crosstalk during bone regeneration process. However, to gather a deeper understanding of these modulatory effects, further investigation including direct osteoclast monoculture as well as hMSCs-osteoclast co-culture on these bioinspired biomaterials are required and suggested as next steps.

In addition to mRNA expression levels of osteogenic biomarkers, we also analyzed ALP activity, as an indicator of osteoblastic function in hMSCs cultured on collagen and biomineralized collagen membranes. ALP is an enzyme found in osteoblasts which increases matrix mineralization (Siffert, 1951; Hessle et al., 2002).

The analysis of the mRNA ALP levels showed that overtime, the ALP expression of hMSCs cultured on TCP control decreased, whereas on collagen and mineralized collagen, no decrease was observed, and the levels at 7 and 14 days were comparable between the two materials. In contrast, an increase in ALP activity was observed between day 3 and day 14, and remained at comparable level until day 21, for hMSCs cultured on both biomimetic membranes as well as the TCP control. This suggests that the peak in the ALP expression at the mRNA level was reached early during the cell culture, and that the effect at the enzyme level is observed at the later time points. Both biomimetic membranes showed a higher ALP activity than the TCP control, with the positive effect of biomineralized collagen being more pronounced at day 3 and day 7. This suggests an enhancing effect of the biomimetic matrices, and in particular the biomineralized one, on osteoblastic activity and, hence, evidence that hMSCs are differentiating into functional osteoblasts.

Previous studies have shown a higher ALP activity of cells cultured on intrafibrillarly mineralized collagen than on collagen without mineral (Jiao et al., 2015; Ye et al., 2016; Zhang et al., 2018) and even on collagen with extrafibrillar mineral (Wang et al., 2018). These findings are in line with ours, although we did not observe significant differences between the two biomimetic membranes. This may be due to differences in the materials used in different studies. Although all studies tested intrafibrillarly mineralized collagen, slight differences in method used for preparing biomineralized collagen could have an impact on important properties of the materials. For example, the extent of exposure of the intrafibrillar mineral from its collagen confinement may be different, affecting both the chemical composition, mechanical properties, and surface topography, which in turn may affect the cell behavior. This is not only the case for the ALP activity but also for expression of other markers of osteogenic differentiation, cell proliferation and ECM production. Therefore, comparisons of different studies that tested biomineralized collagen need to be made with caution.

The formation of cell-induced mineral deposits on collagen and biomineralized collagen membranes was investigated by SEM-EDS. Mineral formation was microscopically observed on the collagen membranes, and supported by the elemental analysis demonstrating the presence of calcium and phosphorus. Critically, calcium and phosphorus were not detected on two controls, i.e., hMSCs cultured on the collagen membranes in GM and collagen membranes incubated in MM medium for 14 days in the absence of cells. These observations strongly suggest that the mineral deposits were indeed produced by the hMSCs cultured in MM, which contains dexamethasone and β-glycerophosphate. Moreover, the morphology of these deposits is interesting, as the mineral appears to surround the collagen fibers. This type of mineralization has been previously described for hMSC undergoing osteogenic differentiation in collagen matrices, where toluidine blue staining allowed identification of mineralized fibers, which were always found in contact with cells (Neuss et al., 2008).

The high content of intrafibrillar CaP mineral in biomineralized membranes made detection of cell-deposited mineral difficult to analyze using SEM-EDS. On SEM images of biomineralized collagen membranes, we did not observe the same type of mineral deposits as seen on collagen membranes. Instead, round particles were observed surrounding the cells, the nature of which is unclear.

While it is reasonable to expect that hMSC cultured on biomineralized collagen in MM would produce mineralized matrix (Langenbach and Handschel, 2013), we did not find convincing evidence for this. One reason for the lack of cell-induced mineral deposits, similar to the ones observed on collagen membranes, may be that the pre-existing intrafibrillar CaP could significantly alter the way in which new mineral is formed, by, for example, prioritizing growth of pre-existing CaP crystals over the formation of new mineral clusters. As existing CaP crystals are inside the collagen fibers, crystal growth would not significantly alter the fibrous morphology of the membrane, consequently making it more difficult to identify “new” mineral using SEM. However, if sufficient new mineral forms, the increase in fiber diameter should be detectable.

While other studies have used Alizarin Red semi-quantification to identify cell-induced mineral deposits (Fu et al., 2016), due to the high content of CaP in our biomineralized collagen membranes, this approach was not successful here (data not shown). Another alternative method for detecting newly formed mineral would be to tag calcium ions in solution with a fluorescent label, which would then be incorporated in newly formed mineral, making detection of deposits possible by fluorescence microscopy (van Gaalen et al., 2010).

Cell-induced matrix mineralization is an important aspect of *de novo* bone formation, and the capacity for hMSCs to mineralize the collagen membrane under stimulation of MM was demonstrated. It is reasonable to expect that hMSCs would show the same behavior when cultured on biomineralized collagen membranes, though we were unable to provide conclusive evidence here.

Taken together, the results of this study that used bioinspired intrafibrillarly mineralized collagen and clinically-relevant hMSCs, have shown that this material supports cell growth and osteogenic differentiation, and also affects markers related to osteoblast-osteoclast crosstalk. Moreover, the ability of the material to support cell-induced mineralization is suggested. While these findings add to the existing knowledge, a few limitations of this study should be discussed. First, the experiments were performed using a single hMSC donor and therefore, donor variability is not taken into account. Second, the induction of osteogenic differentiation was predominantly analyzed at the mRNA level. A next step to support the claim on enhancement of osteogenic differentiation on biomineralized collagen, is to assess the differentiation at the protein level and to further investigate the ECM deposition and mineralization. From a bone regeneration perspective, not only the formation of bone but also its resorption are relevant processes to be considered when developing a bone graft substitute. Our data on osteoblast-osteoclast interaction indicated a possible modulatory effect of both collagen and mineralized collagen on osteoclastic functions and on osteoblast-osteoclast communication, which would be interesting to further explore. Lastly, production of the (mineralized) ECM by hMSCs on biomineralized collagen should be studied further, to clarify the nature of the mineral deposited and how this process is influenced by the properties of the material.

## Conclusion

The results in this study demonstrate that biomineralized collagen membranes support the growth and osteogenic differentiation of hMSCs. The upregulation of the expression of osteogenesis-related genes such as RUNX2, SPP1, ENPP1, OCN, and COL1A1, as well as the enhancement of the ALP activity were observed. Furthermore, biomineralized collagen was shown to affect the expression of osteoclast-related genes OPG and M-CSF. hMSCs were able to deposit mineralized ECM on the collagen membranes and were suggested to have the ability to do the same on biomineralized collagen. We conclude that biomineralized collagen is a promising biomaterial for bone regeneration and merits further study.

## Data Availability Statement

The original contributions generated for this study are publicly available. This data can be found here: https://hdl.handle.net/10411/BC3SR3.

## Ethics Statement

The studies involving human participants were reviewed and approved by the medical ethics committee of Medisch Spectrum Twente (K06-002). The patient provided written informed consent to participate in this study.

## Author Contributions

DM designed the experiments, prepared biomaterials and performed cell culture, DNA and ALP quantification, and SEM-EDS experiments and analyses. ME-L performed qPCR experiments and analyses. DM and ME-L wrote the manuscript. ZB provided helpful insight during manuscript revision. PH supervised the project and reviewed the manuscript. All authors contributed to the article and approved the submitted version.

## Conflict of Interest

The authors declare that the research was conducted in the absence of any commercial or financial relationships that could be construed as a potential conflict of interest.

## Supplementary Material

Supplementary Figure 1 Gene expression profile for RANKL. Osteoblasts in basic medium (BM) and osteoblasts stimulated with 20 nM vitamin D3 (VitD3) were used as controls (Ctrl) to validate the RANKL primers. hMSCs cultured on tissue culture plastic (TCP) control, collagen (Col), and biomineralized collagen (BiominCol) for days 3, 7 and 14 days. Data are represented as mean ± SD (*n* = 2 for BM and VitD3 and *n* = 3 on hMSCs).

Supplementary Figure 2 SEM image of a collagen membrane with hMSCs cultured in GM for 21 days and EDS spectrum of the corresponding area. No calcium and phosphorus was detected.

Supplementary Figure 3 SEM image of a collagen membrane incubated in MM for 14 days in the absence of cells (including media refreshment every 2–3 days) and EDS spectrum of the same area. No calcium and phosphorus signal was detected.
