# Supplementary figures and images for: Proliferation and Osteogenic Differentiation of hMSCs on Biomineralized Collagen
Source: Front Bioeng Biotechnol. 2020 Oct 23;8:554565. doi: 10.3389/fbioe.2020.554565 (PMC7644787; doi:10.3389/fbioe.2020.554565)

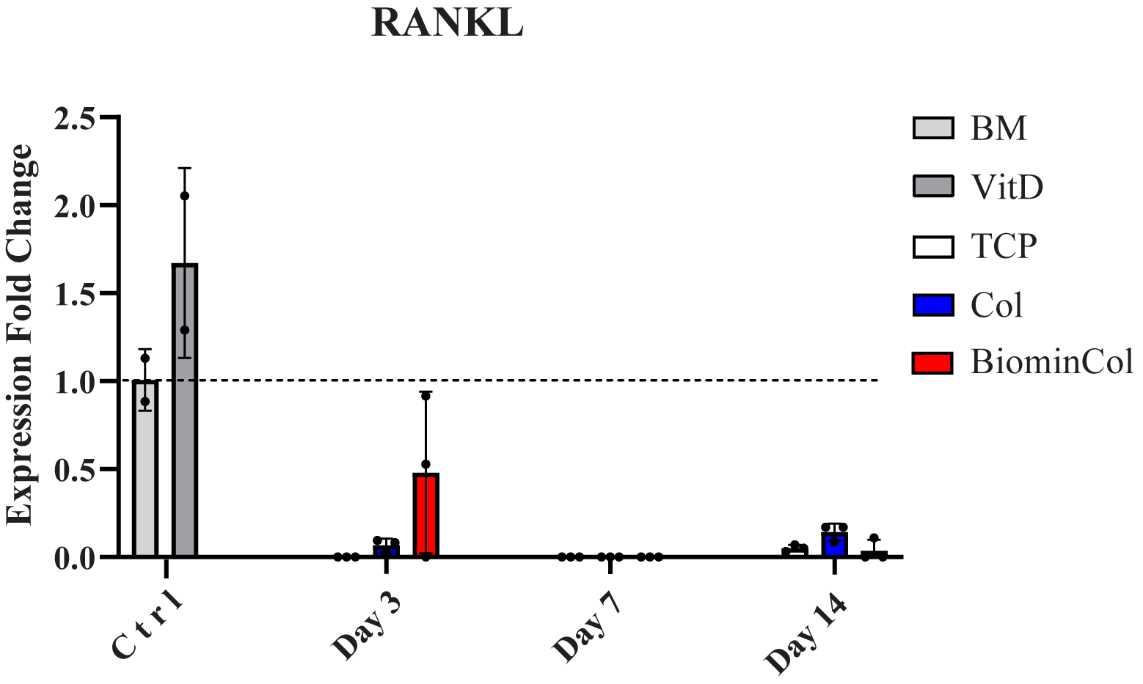

Supplement: Supplementary Figure 1 — Gene expression profile for RANKL. Osteoblasts in basic medium (BM) and osteoblasts stimulated with 20 nM vitamin D3 (VitD3) were used as controls (Ctrl) to validate the RANKL primers. hMSCs cultured on tissue culture plastic (TCP) control, collagen (Col), and biomineralized collagen (BiominCol) for days 3, 7 and 14 days. Data are represented as mean ± SD (n = 2 for BM and VitD3 and n = 3 on hMSCs). [file Image_1.TIF]

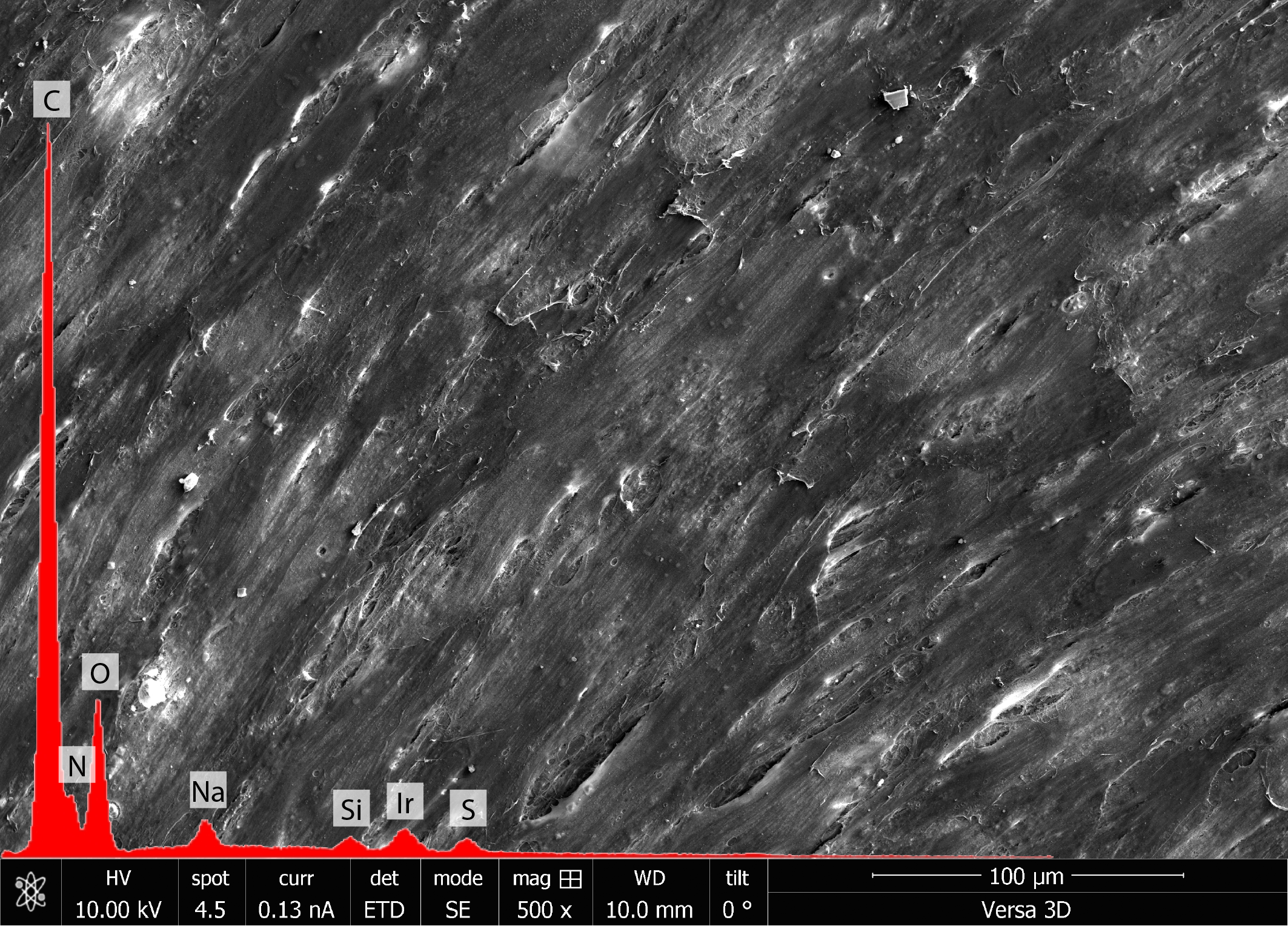

Supplement: Supplementary Figure 2 — SEM image of a collagen membrane with hMSCs cultured in GM for 21 days and EDS spectrum of the corresponding area. No calcium and phosphorus was detected. [file Image_2.TIF]

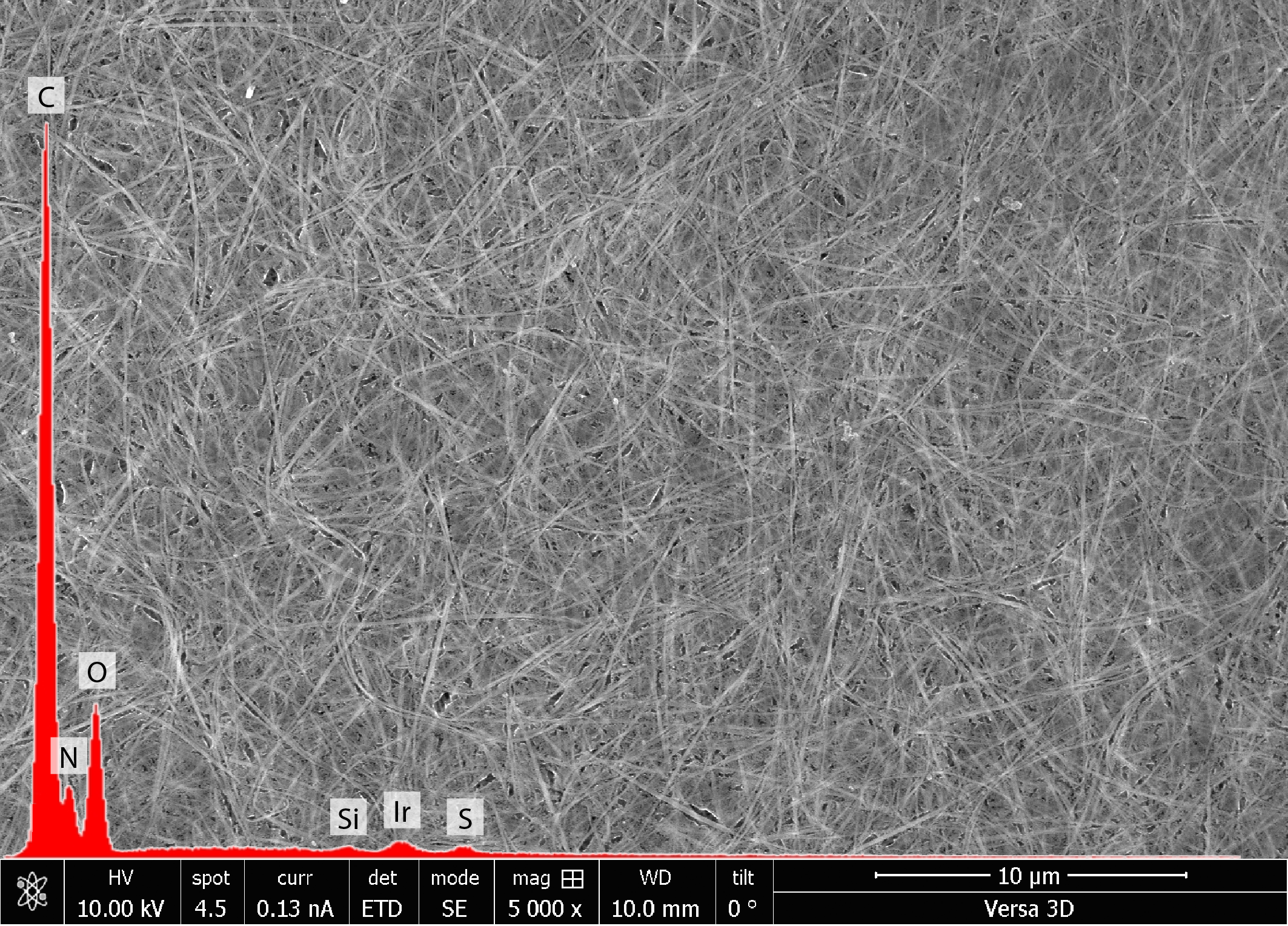

Supplement: Supplementary Figure 3 — SEM image of a collagen membrane incubated in MM for 14 days in the absence of cells (including media refreshment every 2–3 days) and EDS spectrum of the same area. No calcium and phosphorus signal was detected. [file Image_3.TIF]
